# Scalable noninvasive amplicon-based precision sequencing (SNAPseq) for genetic diagnosis and screening of β-thalassemia and sickle cell disease using a next-generation sequencing platform

**DOI:** 10.3389/fmolb.2023.1244244

**Published:** 2023-12-13

**Authors:** Pragya Gupta, V. R. Arvinden, Priya Thakur, Rahul C. Bhoyar, Vinodh Saravanakumar, Narendra Varma Gottumukkala, Sangam Giri Goswami, Mehwish Nafiz, Aditya Ramdas Iyer, Harie Vignesh, Rajat Soni, Nupur Bhargava, Padma Gunda, Suman Jain, Vivek Gupta, Sridhar Sivasubbu, Vinod Scaria, Sivaprakash Ramalingam

**Affiliations:** ^1^ CSIR- Institute for Genomics and Integrative Biology, New Delhi, India; ^2^ Academy of Scientific and Innovative Research (AcSIR), Ghaziabad, India; ^3^ Thalassemia and Sickle Cell Society, Hyderabad, India; ^4^ Government Institute of Medical Sciences (GIMS), Greater Noida, India

**Keywords:** β-thalassemia, sickle cell disease, β-hemoglobinopathies, high-throughput amplicon sequencing, next-generation sequencing, molecular diagnosis

## Abstract

β-hemoglobinopathies such as β-thalassemia (BT) and Sickle cell disease (SCD) are inherited monogenic blood disorders with significant global burden. Hence, early and affordable diagnosis can alleviate morbidity and reduce mortality given the lack of effective cure. Currently, Sanger sequencing is considered to be the gold standard genetic test for BT and SCD, but it has a very low throughput requiring multiple amplicons and more sequencing reactions to cover the entire HBB gene. To address this, we have demonstrated an extraction-free single amplicon-based approach for screening the entire β-globin gene with clinical samples using Scalable noninvasive amplicon-based precision sequencing (SNAPseq) assay catalyzing with next-generation sequencing (NGS). We optimized the assay using noninvasive buccal swab samples and simple finger prick blood for direct amplification with crude lysates. SNAPseq demonstrates high sensitivity and specificity, having a 100% agreement with Sanger sequencing. Furthermore, to facilitate seamless reporting, we have created a much simpler automated pipeline with comprehensive resources for pathogenic mutations in BT and SCD through data integration after systematic classification of variants according to ACMG and AMP guidelines. To the best of our knowledge, this is the first report of the NGS-based high throughput SNAPseq approach for the detection of both BT and SCD in a single assay with high sensitivity in an automated pipeline.

## 1 Introduction

β-thalassemia (BT) and sickle cell disease (SCD) are Mendelian monogenic diseases of red blood cells and are among the most prevalent disorders worldwide. They are caused by genetic mutations in the β-globin (HBB) gene and have an estimated incidence of 40–82 per 1,000 live births (Weatherall et al., 2010; Piel et al., 2013). β-hemoglobinopathies are a prevalent cause of health problems across multiple groups of ethnicities. SCD results from a single amino acid change at the sixth position (G**A**G to G**T**G) in the β-globin gene in which glutamic acid is replaced by valine, whereas more than 200 different mutations are known for BT. These are mainly point mutations or deletions that occur throughout the HBB gene. An estimated 7% of the world’s population carries a defective β-globin gene, and approximately 300,000 babies are born each year with severe hemoglobin abnormalities (Joint WHO-March of Dimes Meeting on Management of Birth Defects and Haemoglobin Disorders, 2006) and recent estimates suggest that this number could increase to more than 400,000 by 2050 (Piel et al., 2017). Treatment options for β-hemoglobinopathies are primarily interventional and do not provide a permanent cure. Hydroxyurea, pain management, and intravenous hydration are used for SCD, whereas frequent blood transfusions coupled with iron chelation therapy are treatment options for BT. Currently, allogeneic hematopoietic stem cell transplantation (HSCT) is the only curative option for both the above available for both the above diseases, but success depends primarily on the availability of human leukocyte antigen (HLA)-matched donor (Ngo and Steinberg, 2015).

Despite advances in the treatment and care of the disease, the incidence rate remains high, which has a profound impact on the public health system and requires significant investment in patient care. Therefore, early detection of the disease remains the realistic approach for disease prevention, reducing the burden on the healthcare system and aiding better disease management with the ultimate goal of reducing patient mortality and morbidity rates. At present, hemoglobin electrophoresis, high-performance liquid chromatography (HPLC) and isoelectric focusing are predominant diagnostic techniques. Generally, HPLC tests require further confirmation by Sanger sequencing-based DNA analysis (Nair, 2018; Arishi et al., 2021). Currently, most commercially available diagnostic methods rely on antibody detection of Hb variants, but these tests may provide deceptive results in blood-transfused individuals. Moreover, commonly used antibody tests in newborn screening, may lead to inconclusive results as they often undergo blood transfusion due to multiple birth-related complications (Whyte and Jefferies, 2014). Hence, subsequent validation after 90 days of transfusion is necessary for such cases (Reed et al., 2000; Kanter et al., 2015). Additionally, the basal level of fetal hemoglobin expression is high in the first few months of the newborn, thus limiting the applicability of antibody-based diagnostic tests. Despite advancement, the genetic diagnosis of BT continues to be challenging due to prevalence of mutations and deletion throughout the HBB gene. Although protein-based techniques can be used to diagnose BT, however, the reliance on HbA2 peak for diagnosis may sometimes lead to misdiagnosis (Nusrat et al., 2011; Yilmaz Keskin et al., 2021). In addition, it cannot identify specific mutations associated with BT (Lama et al., 2022). Hence, a reliable diagnostic method is required that can differentiate the genotype of β-hemoglobinopathies efficiently. Besides early detection of patients, carrier detection will unveil the scope of genetic counseling in reducing the frequency of the disease. World Health Organization (WHO) indexed hemoglobin testing as one of the most crucial *in vitro* diagnostics (IVD) tests for primary care use in developing and underdeveloped countries (Second WHO Model List of Essential *In Vitro* Diagnostics, 2019).

The classification of genetic diseases for accurate diagnosis can be achieved by allele-specific detection of disease mutations. Molecular approaches to identify point mutations are mainly dependent on methods such as the gold standard traditional Sanger sequencing (Ngo et al., 2016) and real-time PCR (Chen et al., 2016) that have very low throughput and require multiple amplicons and several sequencing runs with different primer pairs to cover the entire *HBB* gene and to identify pathogenic variants. In addition, the read quality of Sanger sequencing is often poor in the first 50–80 bases where the primers bind to start amplification. Further, different combinations of compound heterozygous mutations in different ethnic groups demand the need for an integrated diagnosis system that can identify most of the mutations associated with the disease. Moreover, it is challenging to develop a molecular diagnostic test that does not necessitate nucleic acid isolation and purification, as current genotyping methods based on DNA amplification use relatively high DNA purity. Thus, establishing a molecular test that analyzes the samples without DNA extraction and purification has remained elusive. This is carefully addressed in the current study by developing a low-cost, low-complexity buffer system using common laboratory chemicals that enables direct PCR amplification with a simple procedure that effectively releases DNA from buccal swabs or finger prick blood, making sample collection less invasive and efficient.

In recent years, next-generation sequencing (NGS) has become a cost-effective tool for the high-throughput identification of genetic variants, thereby unveiling new opportunities in the field of molecular diagnosis (Korf and Rehm, 2013). When compared to Sanger sequencing, NGS-based amplicon sequencing offers several advantages such as higher sequencing depth with improved sensitivity, enhanced discovery power and higher mutation resolution, high throughput and maximum data that can be generated with the same quantity of DNA. Diagnosis of aneuploidy in embryo biopsy specimens is enabled by this technique, which has been neglected for use in assisted reproductive technology (Fiorentino et al., 2014; Wells et al., 2014; Zheng et al., 2015). Recently, single gene mutations have also been diagnosed in embryos using NGS; however, the techniques used clinically still require extensive customization in the design and optimization of multiplex-PCR for amplification of mutation polymorphisms (Yan et al., 2015; Chen et al., 2016; Ren et al., 2016). Further, Hallem et al. and Haque et al. have validated that NGS in patient-based and population-based carrier screening of recessive inherited disorders and demonstrated acceptable rates of false-positive and false-negative results and the cost-effectiveness of the tool (Hallam et al., 2014; Haque et al., 2016). Besides, NGS has a higher sequencing depth that allows for improved sensitivity and high mutation resolution (Koboldt, 2020), which is not possible with other methods.

In the current pilot study, we presented a unique strategy based on the NGS approach to detect virtually all HBB mutations accountable for SCD and BT. A simple, noninvasive buccal swab specimen or finger prick blood was used, which is economical and can be used as a direct sample matrix avoiding any DNA extraction process. For a proof-of-concept, the robustness of the SNAPseq assay in the detection of allele-specific BT and SCD genotypes from extraction-free non-invasive crude lysates of buccal swab samples with no additional PCR cleanup has been shown with patients, carriers and wild-type samples. A systematic SNAPseq data analysis pipeline was established and validated to prioritize and predict the pathogenicity of all HBB genetic mutations identified in each specimen. The present study shows that a simplified sampling procedure combined with NGS has enormous potential and clinical utility in the molecular diagnosis of genetically heterogeneous diseases such as BT. Our study demonstrated that the NGS-based SNAPseq assay can accurately identify all β-globin mutations, including compound heterozygous variants and large deletions, compared to conventional sequencing approaches. Overall, SNAPseq is a precise and efficient platform for large-scale genetic screening and clinical genotyping in subjects with SCD and BT.

## 2 Materials and methods

### 2.1 Clinical sample collection

The study was approved by the Institutional Ethics Committee at the Government Institute of Medical Sciences (GIMS) Greater Noida and CSIR-Institute of Genomics and Integrative Biology, New Delhi. All the participants provided written informed consent for the study. Peripheral blood samples (collected in EDTA-coated tubes, BD Biosciences) or buccal swab samples or finger prick samples were collected. Buccal swab samples were collected using nylon-flocked buccal swabs (Himedia, India) to swab each inner cheek at least 10–15 times and immediately placed in the lysis buffer.

### 2.2 Sample preparation

Buccal swabs finger pricks direct blood and dry blood spot samples were collected from study subjects and placed in a lysis buffer containing 50 mM NaOH. Each sample was thoroughly mixed for 10 min by vortexing and incubated at 95°C for 10 min. The sample tube was allowed to cool to room temperature, and then the swab was removed from the sample tube. Following this, 120 µL Tris-Cl (pH-8.0) was added to each sample tube to maintain the pH.

### 2.3 Primer design

Multiple primers were designed to amplify the entire HBB gene, covering a range of BT mutations. Primers were designed using the IDT OligoAnalyser tool and synthesized from Sigma-Aldrich, India. All the primers designed are listed in Table 1.

**TABLE 1:** List of primers used in the study.

S.No.	Primer name	Primer sequence (5′-3′)
1	HBB F1	CTA​GGG​TTG​GCC​AAT​CTA​CTC
2	HBB R1	AGT​AAT​GTA​CTA​GGC​AGA​CTG​TG
3	HBB F2	GCA​TCA​GTG​TGG​AAG​TCT​CAG
4	HBB R2	AGG​CAG​AAT​CCA​GAT​GCT​CAA​G
5	HBB F3	TGA​AGG​GCC​TTG​AGC​ATC​TG
6	HBB R3	AGT​TCC​GGG​AGA​CTA​GCA​C
7	HBB F4	TGG​AGA​CGC​AGG​AAG​AGA​TC

### 2.4 Limit of detection

The sensitivity of purified DNA was estimated by cloning the HBB-F4 and HBB-R3 amplicon in the pJET cloning vector and was serially diluted in a range of 10^8^–10^1^ copy number. The copy number was determined by the formula: Number of copies per µL = ((Amount of ds DNA) × (6.022 × 10^23^ molecules per mole))/((length of dsDNA) × 10^9^ ng × (660 g/mol)). Each dilution was used as a template for PCR amplification using HBB-F1 and HBB-R3 primers. For buccal swab and blood samples, a range of 30%–0.1% was used as a template in the total PCR reaction.

### 2.5 PCR amplification for SNAPseq

PCR amplification for the entire HBB gene (includes promoter region, 5′ untranslated region (UTR), all exons, both the introns, and the 3′-UTR) was carried out using various specimens collected from the patients or the volunteers (Genomic DNA, Buccal swabs and Blood) as a template. The amplification was done using PrimeSTAR max polymerase (Takara Bio Inc., Japan) in the recommended concentration. The program set in thermal cycler was 1 min at 98°C as initial denaturation, followed by 35 cycles of 15 s at 98°C for denaturation, 15 s at 58°C for annealing, 15 s at 72°C for extension, and final extension at 72°C for 5 min. The analysis of PCR amplified products was done by electrophoresis on a 1% (w/v) agarose gel and visualized under the Gel Documentation System (Biorad).

### 2.6 PCR amplification and sanger sequencing

Clinical samples were used for HBB gene amplification using three sets of primers to cover the entire HBB gene for Sanger sequencing. The primer combination HBB F1-HBB R1, HBB F2-HBB R2 and HBB F3 -HBB R3 was used for PCR amplification using OneTaq DNA polymerase (NEB, United States). The PCR products were analyzed by agarose gel electrophoresis and column purified with MicroSpin columns (Genetix Biotech, India). The purified samples were subjected to Sanger sequencing using BigDye terminator v3.1 reagents (Thermo Fisher, United States) and carried out at AgrigenomeLabs Pvt. Ltd., Kochi, India. The sequence of the primers is listed in Table 1.

The Sanger sequencing confirmed genotype of the samples were used to determine the sensitivity and the specificity of SNAPseq methodology. The sensitivity and specificity of the assay were calculated as given below;

$$\text{Sensitivity}=\text{TP/}\left(\text{TP}+\text{FN}\right)$$

$$\text{Specificity}=\text{TN/}\left(\text{TN}+\text{FP}\right)$$

where,

TP is True Positive, TN is True Negative, FP is False Positive, and FN is False Negative.

### 2.7 Library preparation and sequencing

The PCR amplified products were subjected to tagmentation, where the product underwent enzymatic fragmentation followed by tagging the fragmented product with the DNA adapter sequences. Limited-cycle PCR was performed on tagmented products to add the Index 1 (i7) adapters, Index 2 (i5) adapters, and sequences required for sequencing cluster generation. The amplified library was purified using the DNA purification beads, followed by quantification using the Qubit High Sensitivity dsDNA quantification kit (Invitrogen). The quality of the final library was assessed by performing agarose gel electrophoresis (1.5%). After the quality assessment, the final libraries were subjected to paired-end sequencing on the Illumina next-generation sequencing platform (MiSeq and iSeq).

### 2.8 NGS data analysis

The amplicon sequencing data was analyzed with an in-house pipeline. Briefly, fastq files were trimmed using trimmomatic (V.0.39) (Bolger et al., 2014), and the resultant fastq files were aligned using bwa mem (V.0.7.17) (Li, 2014) to the human reference genome (GRCh38). Further, the BAM files were sorted, and PCR duplicates were removed using picard Mark Duplicates (V.2.4.1) (Website, 2023). The variants were called using VarScan (V.2.4.4) (Koboldt et al., 2009) and annotated to RefSeq using ANNOVAR (V.2020-06-08) (Wang et al., 2010). Finally, the variants are matched against the ClinVar likely pathogenic/pathogenic list for SCD and BT (ClinVar_20230410.vcf). The entire process of data collection, data analysis, and interpretation as well as the clinical reporting, is envisaged to be automated with little human intervention. The automated pipeline used for data analysis can be found at https://github.com/ARVINDEN/SnapSeq.

## 3 Results

### 3.1 SNAPseq strategy

**FIGURE 1:**
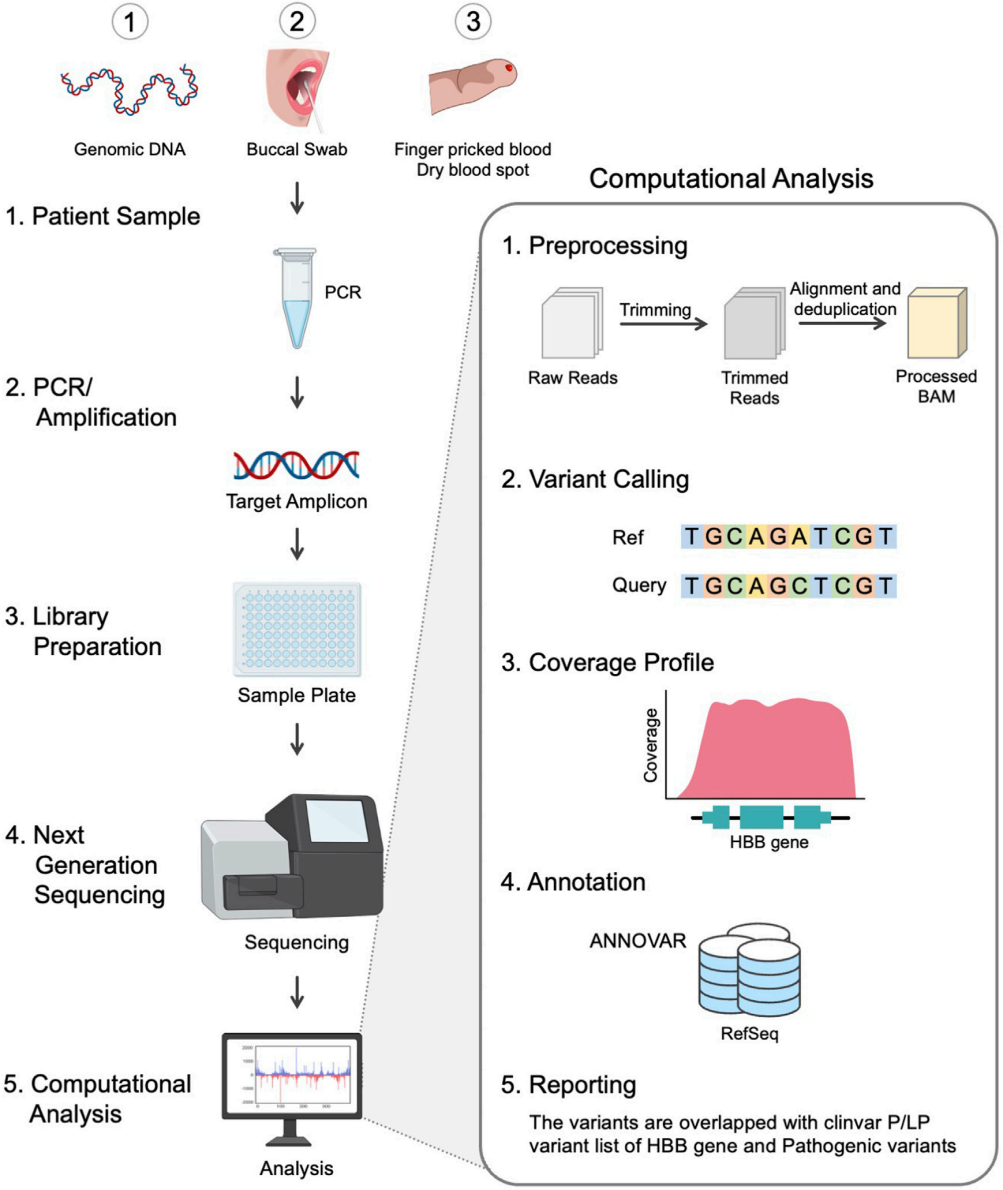
Workflow of the SNAPseq methodology.

The design strategy adopted for the development of scalable noninvasive amplicon-based precision sequencing (SNAPseq) for genetic diagnosis and screening of β-hemoglobinopathies is shown in Figure 1. Clinical samples were collected in the form of blood, a buccal swab or a finger prick. Genomic DNA was isolated from the whole blood, and the other two sets of samples (buccal swabs or finger pricks) were treated with a lysis buffer to release the nucleic acids. PCR amplification was performed to amplify the entire HBB gene as a single amplicon. The PCR products were directly subjected to NGS library preparation as previously described. All the samples were processed in a 96-well plate. Subsequently, 96 samples were pooled together in a single tube. The pooled libraries were denatured and neutralized, and a sequencing run was performed on either the MiSeq or iSeq platforms. The raw data generated in binary base call (BCL) format was analyzed using inhouse developed SNAPseq pipeline (Figure 1). In this study, the entire bioinformatics pipeline, from data collection to clinical reporting, is completely automated to reduce human intervention.

### 3.2 Standardization of primers and polymerases

**FIGURE 2:**
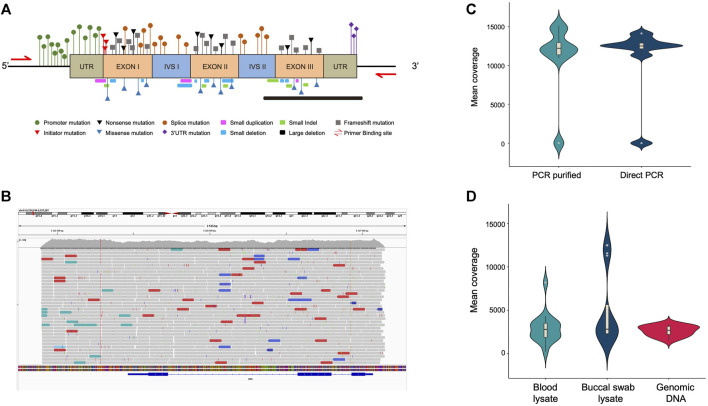
**(A)** Schematic representation of the HBB gene highlighting the BT mutation that spreads throughout the gene. **(B)** Tracks of the amplicon in the integrative genome viewer showing coverage of the entire HBB gene. **(C)** Violin plot showing the mean coverage of PCR purified and direct PCR samples, n = 10. **(D)** Violin plot showing the mean coverage of direct PCR samples amplified from blood lysate, buccal swab lysate and purified genomic DNA, n = 16.

The main objective of the study was to develop a high-throughput integrative assay system that can detect virtually all the mutations associated with SCD and BT in a single amplicon amplified from the HBB gene. In order to enhance the versatility of the assay, designing a primer combination that could efficiently amplify using a wider range of proofreading enzymes was required. To accomplish this, multiple primer combinations spanning the HBB gene were designed. All primer combinations were validated for amplification using multiple proofreading enzymes, including NEBNext, Phusion Polymerase, Amplitaq GOLD, Q5 polymerase, PrimeStar max polymerase, and LA Taq polymerase (Supplementary Figure S1. Our results demonstrated a consistent amplification of the HBB gene using the HBB-F1 and HBB-R3 primer combinations with all the above-mentioned polymerases. The primer combination HBB-F1 and HBB-R3 covers the entire HBB gene, including the promoter, 5′ and 3′ untranslated regions, all three exons, and both introns of the gene (Figures 2A, B). Henceforth, PrimeStar polymerase was used in the study due to its fast extension rate and high fidelity. We further investigated the reliability of NGS outcomes obtained from direct amplicons or column purified amplicons. The elimination of this step would reduce human intervention, reduce cost, and provide a much wider scope for automation. Hence, PCR amplification was performed using genomic DNA obtained from 8 volunteers. Our data indicate a similar median coverage of 12,521.45 and 12,226.15 in direct amplicons and purified amplicons. Hence, these findings indicate the feasibility of direct amplicons for NGS (Figure 2C).

### 3.3 Consistent amplification with different templates

Since the process of genomic DNA isolation is arduous, time-consuming, and expensive, we further investigated whether we could amplify long amplicons using direct lysate prepared from buccal swabs and finger prick blood. This approach would simplify sample collection, expedite the process, and make it ideal for field deployment. Therefore, we attempted to amplify and sequence the HBB gene region from direct blood or buccal swab lysis and compared the coverage with genomic DNA. We observed a consistent amplification of the HBB gene with selected primer combinations in all three template types obtained from 8 volunteers (Supplementary Figure S2). Further, targeted sequencing resulted in similar median coverage of 2,695.04, 2,827.79, and 2,803.20 in amplicons obtained using purified genomic DNA, buccal swab lysate, and blood lysate respectively, as shown in Figure 2D. This shows the use of direct blood lysate or buccal swab lysate as a template, that reproduces similar results and hence would be useful to reduce cost and time.

### 3.4 Limit of detection

**FIGURE 3:**
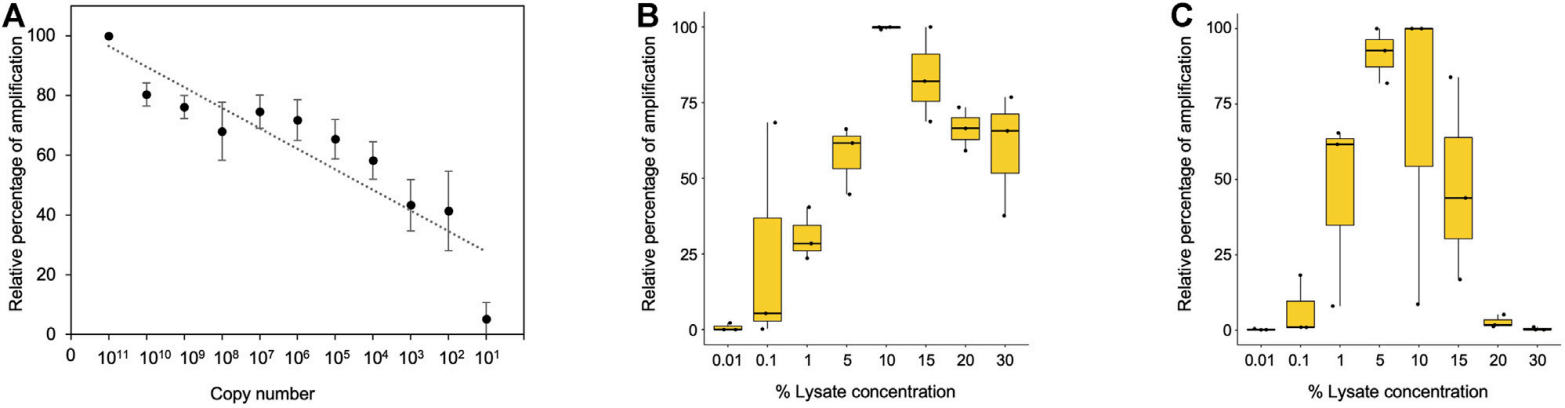
**(A)** Minimum copy number required for amplification with purified plasmid DNA. Densitometric analysis was performed using ImageJ software, and data from dilution 10^11^ were considered as 100%. Remaining dilutions were plotted as percentage relative to 100% (10^11^ dilution), n = 3. **(B)** Boxplot showing the minimum and maximum percentage of blood lysate required for amplification. Densitometric analysis was performed using ImageJ software, and the highest data value was considered 100%. The remaining samples were plotted as percentage relative to 100% (10^11^ dilution), n = 3. **(C)** Boxplot showing the minimum and maximum percentage of buccal swab lysate required for amplification. Densitometric analysis was performed using ImageJ software, and the highest data value was considered as 100%. The remaining samples were plotted as percentage relative to 100% (10^11^ dilution), n = 3.

Further, we investigated the limit of detection for all three template types (purified genomic DNA, buccal swab lysate, and blood lysate). In order to assess the limit of detection in purified DNA samples, a range of 10^8^–10^1^ copies per microliter dilution of pHBB plasmid was used. The results suggested that polymerase is efficient enough to amplify as few as 10 target DNA copies (Figure 3A, Supplementary Figure S3A). Direct lysates contain genomic DNA in crude form; hence, it becomes challenging to calculate the copy number accurately. To address this issue, we conducted additional tests to determine the maximum percentage of direct lysate that could potentially hinder the PCR reaction due to the presence of salts. Additionally, we also tested the minimum amount of lysate required for amplification. To achieve this, we performed PCR using a range of 30%–0.01% of direct lysate as a template obtained from 3 volunteers. Our results indicate that as much as 30% of direct blood lysate was not inhibitory for PCR amplification (Figure 3B, Supplementary Figure S3B). On the other hand, 20% of buccal swab lysates in the PCR reaction were inhibitory, possibly due to the presence of fibers from the buccal swab that may have been left behind during processing (Figure 3C, Supplementary Figure S3C). Notably, a much higher variation in amplification was observed in buccal swab lysate as compared to blood lysate due to individual collection variability. Hence, the amount of genomic DNA may vary from individual to individual due to sampling variability. Nevertheless, our data demonstrates that as low as 0.1% of both lysates was sufficient for amplification (Figures 3B, C, Supplementary Figures S3D, E). However, in the clinical setting, variations in sample collection could arise due to inter-individual variability, hence, a range of 1%–15% of direct lysates is recommended to amplify from direct lysates.

### 3.5 Effect of time and temperature on crude genomic DNA lysates

**FIGURE 4:**
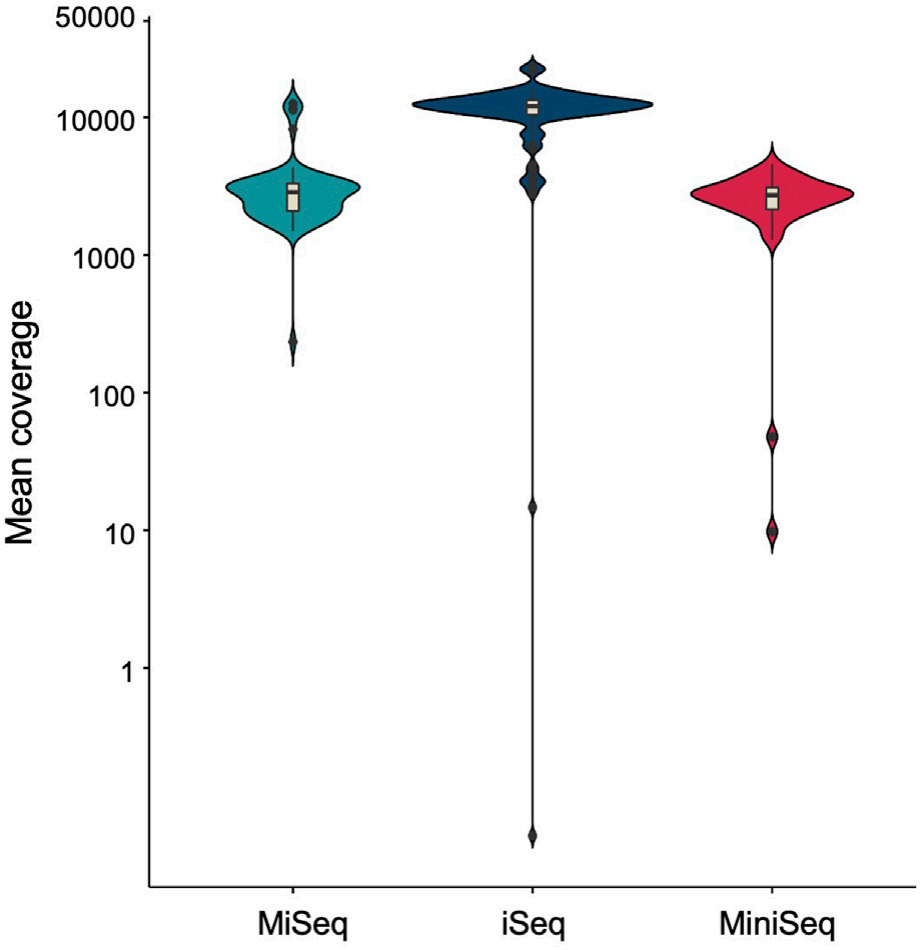
Violin plot showing mean coverage of MiSeq and iSeq sequencing platforms.

As buccal swab lysates are non-invasive and can serve as a source of crude genomic DNA, we further evaluated the stability of unprocessed buccal swab samples under variable temperatures. We have demonstrated previously that storing buccal swab samples at different temperatures (25°C, 37°C, and 42°C) prior to processing results in consistent amplification for short amplicons (Thakur et al., 2022). We adopted the same methodology to assess its impact on the amplification of large amplicons. The buccal swab samples were collected and stored at all three temperatures for 5 days, followed by further processing and PCR amplification. Our results indicated consistent amplification with buccal swab sample lysates at all different temperatures (Supplementary Figure S4A) (Table 2). To further extend the scope of sample availability, we tested if dry spots of blood could also be used as a genomic source. In a similar manner to buccal swab samples, dry spots of blood samples were stored at 25°C, 37°C, and 42°C for 5 days. Afterwards, lysates were prepared, and our amplification data demonstrated consistent amplification with all the samples (Supplementary Figure S4B) (Table 2). In summary, our findings indicate that both buccal swabs and blood (dry spot) lysates result in consistent amplification for NGS. We further assessed the sensitivity and specificity of sequencing by employing three well known bench-top sequencing platforms: MiSeq, iSeq and MiniSeq. Our data demonstrated 2,848.78, 12,010.85 and 2,690.19 median coverage for MiSeq, iSeq and MiniSeq respectively (Figure 4). This indicates a wider application of our assay system.

**TABLE 2:** A comparison of PCR amplification using direct lysates stored at different temperature for 5 days.

Sample type	Storage temperature (°C)	Number of days of storage	Sensitivity
Buccal swab	25	5	6/6
	37	5	6/6
	42	5	6/6
Blood (dry spots)	25	5	5/5
	37	5	5/5
	42	5	5/5

### 3.6 Comprehensive and precise identification of genetic mutations associated with β-hemoglobinopathies

**FIGURE 5:**
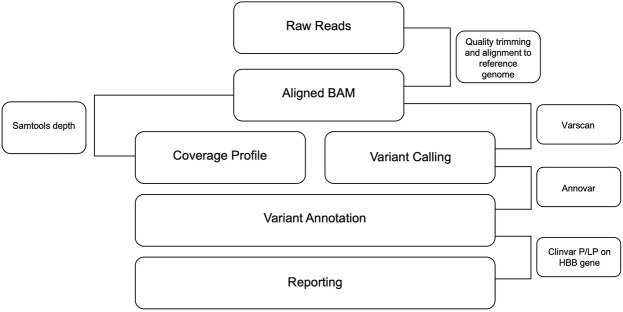
Flowchart depicting the pipeline for CD and BT mutation identification. Sequencing data were analyzed using the in-house pipeline. Fastq files were trimmed using the trimming algorithm, and the resulting Fastq files were aligned to the human reference genome. PCR duplicates were then removed and variants were called using Varscan and annotated to RefSeq using Annovar. Finally, variants were aligned with the Clinvar list of likely pathogenic/pathogenic variants for SCD and BT, which is the output of this pipeline.

Next, to validate the accuracy of our pipeline for precisely identifying the genetic mutation associated with β-hemoglobinopathies (Figure 5), we collected samples in the form of blood (for genomic DNA isolation), buccal swab or finger prick blood. We ensured coverage of the BT mutation present in the different regions of the gene as well as the compound heterozygous variants, including HbS/BT, BT/BT. We amplified the target region for over 100 samples with known mutations and found that the results generated by our pipeline were in concordance with the Sanger sequencing results. This indicates that SNAPseq is a highly sensitive and specific method of mutation detection (Table 3). We further sought to test the genotype of patients clinically declared to have thalassemia based on their clinical profile. We were able to identify the mutation in the patients. Further, our data indicate that out of all BT patients, 25% were compound heterozygous for the mutations. All the different mutations from known and unknown samples identified using the SNAPseq methodology for this study are listed in Table 4. The critical steps involved in SNAPseq methodology is listed in Supplementary Table S1.

**TABLE 3:** Sensitivity and specificity of SNAPseq method.

S.No.	Genotype	Number of samples	True positives	False negative	True negative	False positive
1	Wild Type (HbA/HbA)	13	0	0	13	0
2	Sickle cell trait (HbS/HbAA)	10	10	0	0	0
3	Sickle cell disease (HbS/HbS)	39	39	0	0	0
4	β-thalassemia	36	36	0	0	0
5	Compound heterozygous or heterozygous	7	7	0	0	0
	**Total**	**105**	**92**	**0**	**13**	**0**

Sum of all the participant in respective categories have been bold.

**TABLE 4:** List of mutations identified in the study.

S.No.	Disease	Common name	Chromosome location	HGVS name
**β-thalassemia**				
**1**	β-thalassemia	IVS I-5 (G>C)	Chr11-5226925	HBB:c.92 + 5G>C
**2**	β-thalassemia	CD 8/9 (+G)	Chr11-5226994	HBB:c.27dupG
**3**	β-thalassemia	CD 26 GAG>AAG	Chr11-5226943	HBB:c.79G>A
**4**	β-thalassemia	CD 41/42 (-CTTT) (CD 41/42 (-TTCT), CD 41/42 (-TCTT)	Chr11-5226762	HBB:c.126_129delCTTT
**5**	β-thalassemia	CD 17 (+A)	Chr11-5226970	HBB:c.53dup
**6**	β-thalassemia	CD 15 TGG>TAG	Chr11-5226975	HBB:c.47G>A
**7**	β-thalassemia	CD 5 -CT	Chr11-5227003	HBB:c.17_18delCT
**8**	β-thalassemia	IVS I-1 (G>C)	Chr11-5226930	HBB:c.92 + 1G>C
**9**	β-thalassemia	619 bp deletion (Asian Indian)	Chr11-5225256-5225875	NG_000007.3:g.71609_72227del619
**10**	β-thalassemia	IVS I-1 G>A	Chr11-5226930	HBB:c.92 + 1G>A
**11**	β-thalassemia	Init CD ATG>ACG	Chr11-5227020	HBB:c.2T>C
**Compound heterzogotes (β thalassemia/β-thalassemia)**				
**1**	β-thalassemia	IVS I-5 (G>C)/IVS I-1 (G>C)	Chr11-5226925/Chr11-5226930	HBB:c.92 + 5G>C/HBB:c.92 + 1G>C
**2**	β-thalassemia	CD 26 GAG>AAG/CD 15 TGG>TAG	Chr11-5226943/Chr11-5226975	HBB:c.79G>A/HBB:c.47G>A
**3**	β-thalassemia	IVS I-5 (G>C)/CD 15 TGG>TAG	Chr11-5226925/Chr11-5226975	HBB:c.92 + 5G>C/HBB:c.47G>A
**4**	β-thalassemia	IVS I-5 (G>C)/IVS II-1 G>A	Chr11-5226925/Chr11-5226576	HBB:c.92 + 5G>C/HBB:c.315 + 1G>A
**5**	β-thalassemia	IVS I-5 (G>C)/CD 41/42 (-CTTT) (CD 41/42 (-TTCT), CD 41/42 (-TCTT))	Chr11-5226925/Chr11-5226762	HBB:c.92 + 5G>C/HBB:c.126_129delCTTT
**6**	β-thalassemia	IVS I-5 (G>C)/CD 54 -T	Chr11-5226925/Chr11-5226725	HBB:c.92 + 5G>C/HBB:c.165delT
**7**	β-thalassemia	IVS I-5 (G>C)/IVS II-837 (T>G)	Chr11-5226925 Chr11-5225740	HBB:c.92 + 5G>C/HBB:c.316-14T>G
**8**	β-thalassemia	VS. I-1 (G>C)/CD 8/9 (+G)	Chr11-5226930/Chr11-5226994	HBB:c.92 + 5G>C/HBB:c.27dupG
**Compound heterozyotes for HbS/β-thalassemia**				
**1**	HbS/β-thalassemia	CD 6 (GAG>GTG)/IVS I-5 (G>C)	Chr11-5227002/Chr11-5226925	(HBB):c.20A>T/HBB:c.92 + 5G>C
**2**	HbS/β-thalassemia	CD 6 (GAG>GTG)/41/42 (-CTTT) (CD 41/42 (-TTCT), CD 41/42 (-TCTT))	Chr11-5227002/Chr11-5226762	(HBB):c.20A>T/HBB:c.126_129delCTTT
**3**	HbS/β-thalassemia	CD 6 (GAG>GTG)/619bp deletion	Chr11-5227002/Chr11-5225256-5225875	(HBB):c.20A>T/NG_000007.3:g.71609_72227del619
**Sickle cell disease (HbSS)**				
**1**	Sickle cell disease (HbS/HbS)	CD 6 (GAG>GTG)	Chr11-5227002	(HBB):c.20A>T/(HBB):c.20A>T
**2**	Sickle cell trait (HbS/HbA)	CD 6 (GAG>GTG)/WT	Chr11-5227002	(HBB):c.20A>T/WT

In order to evaluate the robustness and reproducibility of the SNAPseq assay, we assessed the above protocol at a secondary healthcare center, where sample collection, sample processing, PCR amplification, NGS library preparation, and sequencing run were performed by the laboratory technicians and good quality data was obtained. This clearly demonstrates the reproducibility and robustness of the assay and how easily it can be adopted in a healthcare setting for high-throughput screening.

## 4 Discussion

Hemoglobinopathies are the most common monogenic disorders and impose a significant global health burden on families and the healthcare system across the globe. Hence, the early diagnosis of hemoglobinopathies is imperative for both prevention and appropriate treatment for patients. Despite extensive studies at the biochemical, molecular, and hematological levels, the differential diagnosis of hemoglobinopathies remains challenging.

The National Institute for Health and Care Excellence (NICE) has recommended for sickle cell/trait testing in the panel of preoperative tests (Routine preoperative tests for elective surgery: ^©^ NICE, 2016; Walker et al., 2021). Their high prevalence rate makes them indispensable tests in newborn screening. At present, the diagnosis of β-hemoglobin disorders is an extensive process involving several stages of screening: Evaluation of the blood panel and specialized hemoglobin tests for characterization. Most antenatal screening and newborn screening (NBS) programs utilize protein-based hemoglobin separation techniques such as isoelectric focusing (IEF), HPLC, and gel- or liquid-based electrophoresis (Ryan et al., 2010; Therrell et al., 2015). Although protein-based techniques form the basis of hemoglobin diagnostics, they may not be able to identify BT mutations or distinguish between HbSS and compound heterozygosity for HbS (Belhoul et al., 2013; Shook et al., 2021). They are also time consuming and additional confirmatory tests are necessary to confirm diagnosis which delays point-of care. Therefore, to avoid misleading results additional confirmation of the mutations by DNA sequencing is required (Belhoul et al., 2013). Low MCH (mean corpuscular hemoglobin) and MCV (mean corpuscular volume) are common symptoms in hemoglobinopathies, but these symptoms may often be misdiagnosed for vitamin B12, iron, and folic acid deficiencies (Prueksaritanond et al., 2013). If β-hemoglobinopathy with the above symptoms is suspected, a series of specialized hemoglobin tests is recommended. Thus, multiple diagnostic steps may lead to misdiagnosis, delayed treatment, and overlooking of mutation carriers requiring counseling. There are multiple literature reporting pitfalls of currently used for hemoglobinopathies screening programs in prevention and therapy of hemoglobinopathies (Belhoul et al., 2013; Shook et al., 2021). In addition, the diagnostic differentiation is now time-sensitive and crucial due to the rising use of early, pre-symptomatic disease-modifying medication (e.g., initiation of hydroxyurea therapy at 6 months of age) (Ware et al., 2019).

The rising prevalence of compound heterozygotes of BT and SCD presents an additional challenge for point-of care or simple molecular-based genetic testing. Similar to the variable phenotypes associated with BT, compound heterozygosity of BT and SCD results in a variable clinical presentation depending on the mutation. Approximately 10%–15% of sickle cell disease is caused by a combination of the SCD and BT mutations, also known as compound heterozygosity (Aygun et al., 2022), which cannot be detected by point-of-care screening methods. The distinction between HbSS and HbS/β genotypes using current hemoglobin-based diagnostic methods can be difficult (Chandra et al., 2017; Uçucu et al., 2022) and thus there is a need for a scalable and comprehensive genetic diagnostic method that can identify virtually all HBB mutations associated with β-hemoglobinopathies.

NGS has transformed genomics research and developed better sequencing methods. Although sanger sequencing is competent for sequencing amplicons up to 1 kb (Heather and Chain, 2016), screening longer templates require primer walking or shotgun sequencing, which are more complicated and expensive (McMurray et al., 1998; Emonet et al., 2007). The ability of SNAPseq to evaluate long amplicons is a significant advantage in such cases. SNAPseq also has a notable advantage over Sanger sequencing in that it often provides full coverage for the target amplicon. Generation of entire coverage through Sanger sequencing requires bidirectional analysis to avoid the interpretational complications brought on by “dye blobs” and ambiguity in base calling at the start of each sequence, which can vary from 60 to 120 bases of the sequence (Kieleczawa, 2005). Although Sanger sequencing can sequence up to 1 kb, the quality of 1 Kb reads would only be about 700–800 bp due to the reason mentioned above.

Advancements in the field of NGS and its deep sequencing to identify mutations and variants have fast-tracked the diagnosis of many human genetic disorders and have been more reliable in characterizing the disease genotype than other molecular diagnostic tests available (Haque et al., 2016). In recent years, substantial advancements in this field have led to variety of applications in research and genomic medicine. Recently, Kubikova et al. utilized a Next-Generation Sequencing (NGS) approach to diagnose β-hemoglobinopathies in pre-implantation embryos (Kubikova et al., 2018). In this study, 8 pairs of primers were used to amplify 12 different PCR fragments spanning the coding regions of the HBB gene and splice acceptor and donor sites using purified genomic DNA extracted from participant samples. There are many BT mutations that are present in the intronic regions that are not completely covered in the study, and the amplification of multiple fragments with different primer sets using purified genomic DNA is an expensive, time-consuming, and cumbersome process, thus limiting its application for scalable diagnosis. Another study by He et al. reported thalassemia carrier screening using an NGS-based approach, but the study was designed to identify only the most common disease-causing point mutations or selected regions (He et al., 2017).

Although NGS-based molecular diagnostics have been successfully used for various genomic applications (Taylor et al., 2020) extraction-free, universal single-amplicon sequencing strategy to identify all HBB mutations with an automated pipeline has not yet been developed. In this pilot study, we developed and optimized a SNAPseq methodology using crude lysates of buccal swabs and/or blood and analyzed them using an established pipeline to prioritize pathogenic mutations with allele-specific sensitivity. The SNAPseq pipeline is designed to identify both common and rare, annotated and novel variants in carriers with and without BT and sickle cell trait phenotypes, thus significantly improving carrier screening and subsequently improving the detection rate of at-risk couples.

In developing the SNAPseq assay system, one of the main challenges was to address the method of sample collection and processing, so that the samples are collected in the quickest and most efficient way without compromising the accuracy and sensitivity of the test. Blood samples have been traditionally used for molecular studies and clinical diagnostics. Nevertheless, blood collection for genomic DNA isolation is an invasive procedure that demands skilled healthcare professionals (Javaid et al., 2016). Developing a genetic test that does not require isolation and purification of genomic DNA is challenging because existing molecular tests based on DNA amplification require relatively high purity of genomic DNA (Thakur et al., 2022). Therefore, the development of a molecular test that can analyze the samples without the need for isolating genomic DNA isolation has proven to be challenging. In this study, we developed a low-complexity buffer system using commonly available laboratory reagents that is both cost-effective and simple to use. This system enables direct amplification of genomic DNA through a straightforward protocol that maximizes the release of DNA. We utilized the crude lysate prepared from a simple finger prick blood, which does not require professional assistance and also results in consistent amplification. Furthermore, saliva is a more suitable and minimally intrusive alternative to blood for molecular diagnostics. While saliva collection can be challenging due to the common medication side effect of dry mouth, however, buccal swab sample collection is rapid, non-invasive, and inexpensive cost-effective. We have further compared PCR products from our extraction-free protocol to PCR products amplified using purified genomic DNA obtained from the same samples. We found a similar outcome between the two methods.

The maintenance of sample integrity is crucial, especially when samples are being transported over longer distances. Ideally, biological samples should be stored in temperature-controlled and properly qualified storage and processed as quickly as possible after collection (Vaught and Henderson, 2011). Hence, sample storage is another obstacle during sample collection since most of the collected samples need to be stored and transported before processing in a realistic scenario. Consequently, we have demonstrated that buccal swab samples in the collection buffer are stable at 25°C, 37°C, and 42°C for up to 5 days. In addition, our study also demonstrated that finger-prick blood samples are also stable for up to 5 days as dry spots. Our study highlights the crucial significance of our findings for easy adoption of the SNAPseq assay in low-and middle-income nations with a high prevalence of β-hemoglobinopathies. These countries often face limited access to expensive refrigerated transport and adequate storage facilities for biological samples in remote areas.

An additional advantage of the SNAPseq assay system is that it eliminates the need for extra PCR cleanup steps during downstream applications, such as NGS. These crucial optimizations made for the SNAPseq assay system not only leads to significantly reduced cost but also makes it highly suitable for adoption in high throughput settings. Furthermore, sample collection and transportation for the SNAPseq system is easily field-deployable. Patients and their families can conveniently collect their samples at homes or clinics, which can then be transported to a higher resource setting with access to an NGS facility. In addition, in this study, we evaluated the ability of three of the major Illumina sequencing platforms, iSeq, MiniSeq, and MiSeq, which covers both high-throughput and low-throughput samples, and obtained similar results. Our optimized assay pipeline system provides clinically relevant sensitivity in mutation calling across the HBB gene.

In this study, we also provide a general workflow for the interpretation of the enormous amount of data produced through NGS-based molecular diagnosis. Using an SNAPseq approach, we analyzed over 250 samples with high sensitivity using a single amplification covering virtually all the HBB mutations with a simple and extraction-free platform. In addition to SCD and BT mutations, SNAPseq can identify other genotypes such as hemoglobin type C genotypes AC, CC (HBC), and SC (compound heterozygous for HbC and HbS) and hemoglobin E (HBE) BT mutation (point mutation in codon 26 GAG to AAG) on the first allele and any other BT mutation in the second allele. Here, we have outlined a highly sensitive NGS-based carrier screening system that has a number of advantages over conventional genotyping techniques and exhibits excellent levels of precision, reproducibility, and resilience for clinical usage. The immense potential and clinical use of NGS-based SNAPseq are emphasized by the high diagnostic yield attained by the identification of genetic mutations of all forms of inheritance, particularly *de novo* mutations.

A similar diagnostic workflow can also be easily adopted for screening other genetic diseases with very little customization. It can offer a quick, simple, and potentially affordable high-throughput approach for screening genetic disease to detect a wide range of genotypes, including point mutations, large deletions, and indels. It provides a great opportunity for better assessment, disease management, and genetic counseling.

Despite several advantages, the current primer set would not enable the detection of rare large deletions in the β-gene cluster, δβ-thalassemia mutations and α-thalassemia mutations. Nonetheless, the comprehensive SNAPseq approach facilitates the diagnosis of this condition by enabling multiplexing of this region would further aid in the diagnosis of such conditions.

In summary, we developed a highly sensitive, comprehensive, and precise molecular diagnostic tool called SNAPseq, which is simple and could be scaled up for population level screening and systematic analysis and interpretation of all identified genetic mutations for β-hemoglobinopathies, a most common monogenic disease. This could be of high potential use in regions where the prevalence of β-hemoglobinopathies is high and this upscale adoption could serve as a gold standard technique applicable for precise diagnosis of β-hemoglobinopathies.

## Data Availability

The original contributions presented in the study are included in the article/Supplementary Material, further inquiries can be directed to the corresponding authors.
