# Supplementary material for: Scalable noninvasive amplicon-based precision sequencing (SNAPseq) for genetic diagnosis and screening of β-thalassemia and sickle cell disease using a next-generation sequencing platform
Source: Front Mol Biosci. 2023 Dec 13;10:1244244. doi: 10.3389/fmolb.2023.1244244 (PMC10751313; doi:10.3389/fmolb.2023.1244244)
Supplement: Supplementary file 1 [file Table1.DOCX]

| **Procedure** | **Critical steps** |
| --- | --- |
| Sample Collection | Best practice for buccal swab collection has to be followed to ensure sufficient DNA sample quantity, recommending vigorous swabbing for at least 10 seconds from inside of each cheek. |
| Sample processing | 1. In the clinical setting, variations in sample collection could arise due to inter-individual variability, hence, a range of 1%-15% of direct lysates is recommended for amplification. However, in some cases, the amplification may be hindered while using crude lysates due to the presence of a very high or low amount of genomic content. In such scenarios, the amount of lysate should be decreased or increased accordingly.  2. The recommended storage of direct lysate is 4°C and can be stored for 3-6 months. Repeated  cycles of room temperature and subsequent cooling to 4°C of samples may affect its quality and therefore PCR amplification. |
| Library preparation | 1. Incomplete fragmentation in tagmentation-based library preparation can lead to biases, lower library complexity, PCR amplification issues, and challenges in downstream analysis. To mitigate these limitations, optimizing the tagmentation protocol to achieve complete and uniform fragmentation is crucial. Quality control steps, such as assessing fragment size distribution, can help identify and address issues related to incomplete fragmentation.  2. Library quantification is crucial as it determines the library's concentration accurately. Loading the correct library concentration is essential for optimal cluster generation during sequencing. Insufficient or excessive library amounts can lead to suboptimal sequencing results, affecting data quality and analysis. Precise quantification ensures the success of the sequencing run and minimizes data variability. Hence fluorescence-based DNA quantification such as Qubit  is recommended. |
| Data analysis pipeline | 1.Routine quality checks of NGS data analysis should be followed like base quality, target coverage etc.  2. Always use the latest version of ClinVar VCF with the correct reference build (hg19/hg38). Additionally, it is  important to re-check   the VCF file when no pathogenic mutation is reported in the final table, as rare unreported mutations might be missed from overlapping with ClinVar pathogenic or likely pathogenic variants. |

**Supplementary Table 1:** Critical steps involved of SNAPseq methodology.
