# Supplementary material for: Scalable noninvasive amplicon-based precision sequencing (SNAPseq) for genetic diagnosis and screening of β-thalassemia and sickle cell disease using a next-generation sequencing platform
Source: Front Mol Biosci. 2023 Dec 13;10:1244244. doi: 10.3389/fmolb.2023.1244244 (PMC10751313; doi:10.3389/fmolb.2023.1244244)
Supplement: Supplementary file 2 [file DataSheet1.PDF]

Scalable non-invasive amplicon-based precision sequencing (SNAPseq) for genetic diagnosis and screening of β-thalassemia and sickle cell disease using next-generation sequencing platform

Supplementary Figures

Supplementary Fig. 1

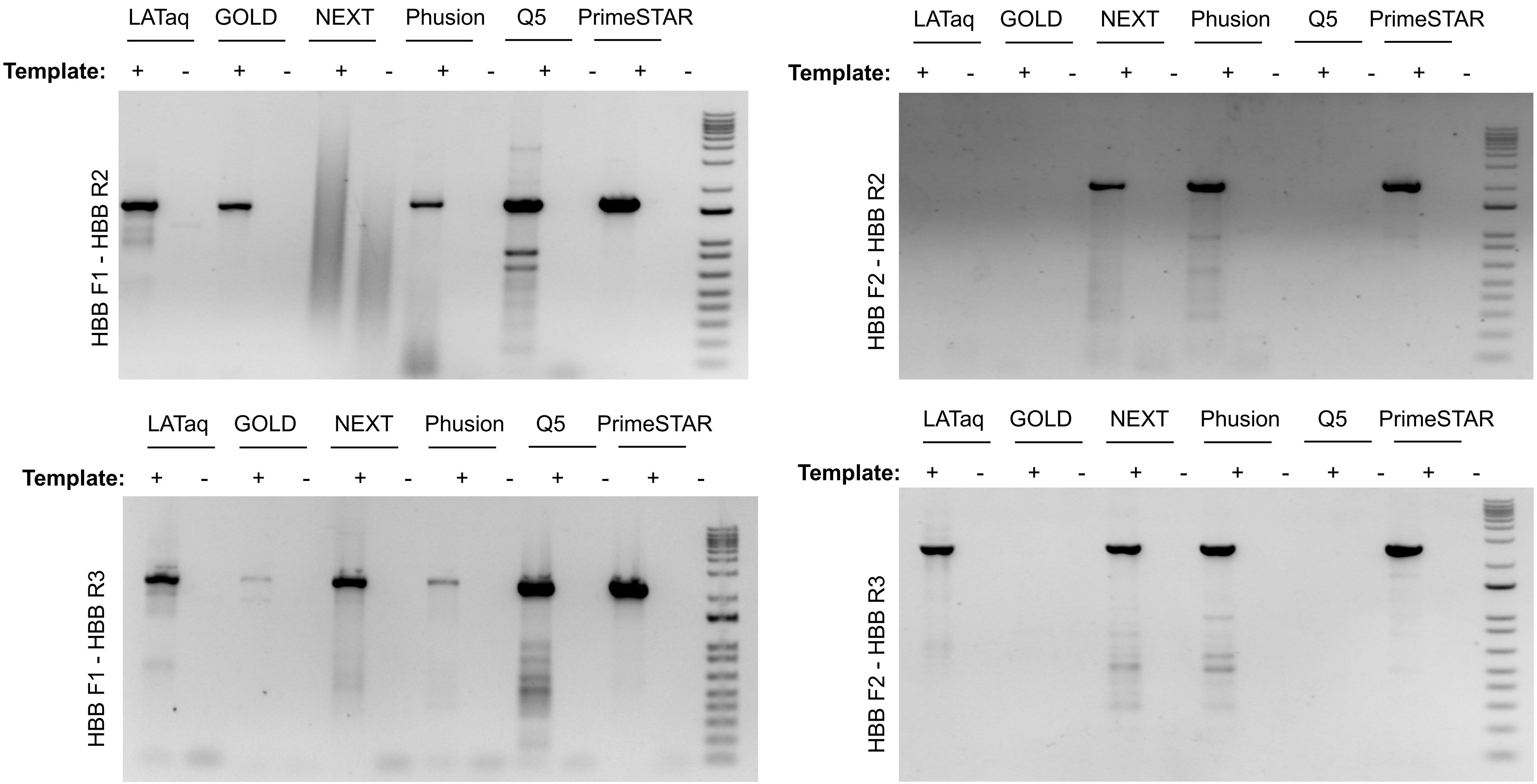

Supplementary Figure 1: PCR amplification of HBB gene using different primer combination and proofreading polymerases.

Supplementary Fig. 2

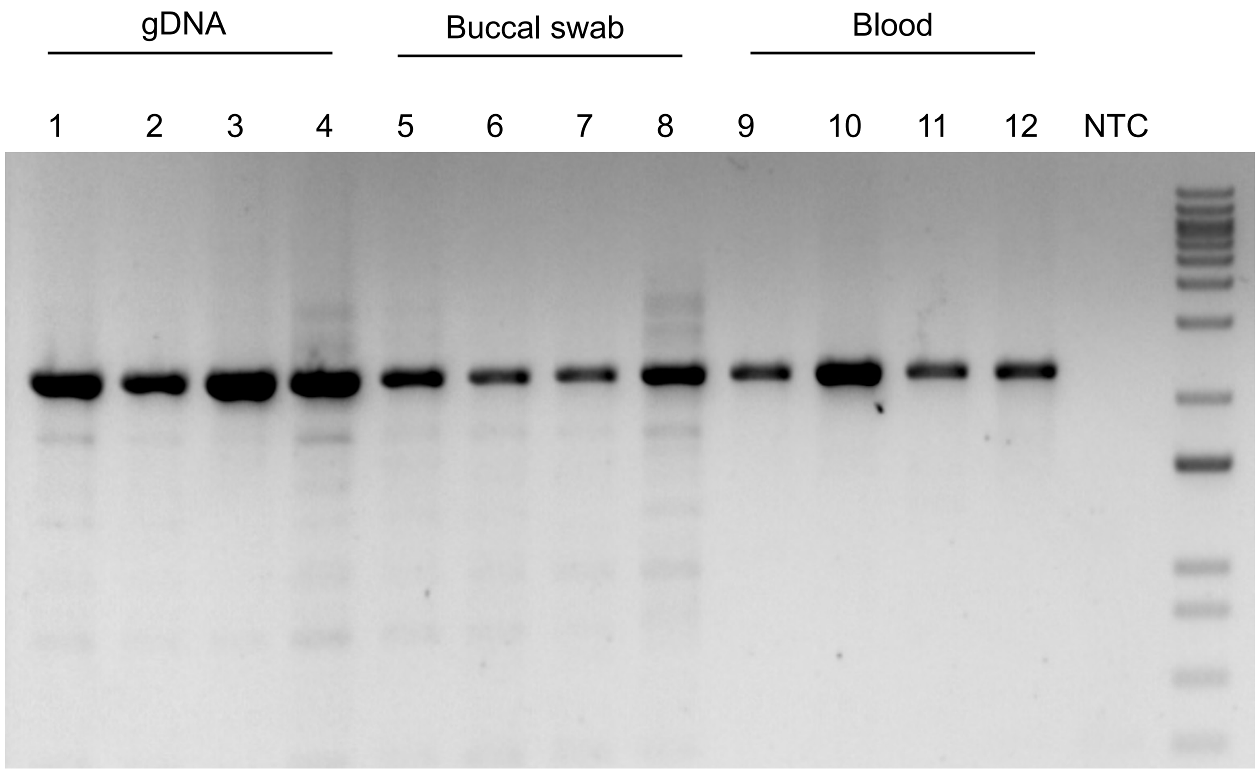

Supplementary Figure 2: Representative agarose gel image demonstrating amplification of HBB genes from 4 volunteers using purified genomic DNA (gDNA), buccal swab lysate and blood lysate

Supplementary Fig. 3

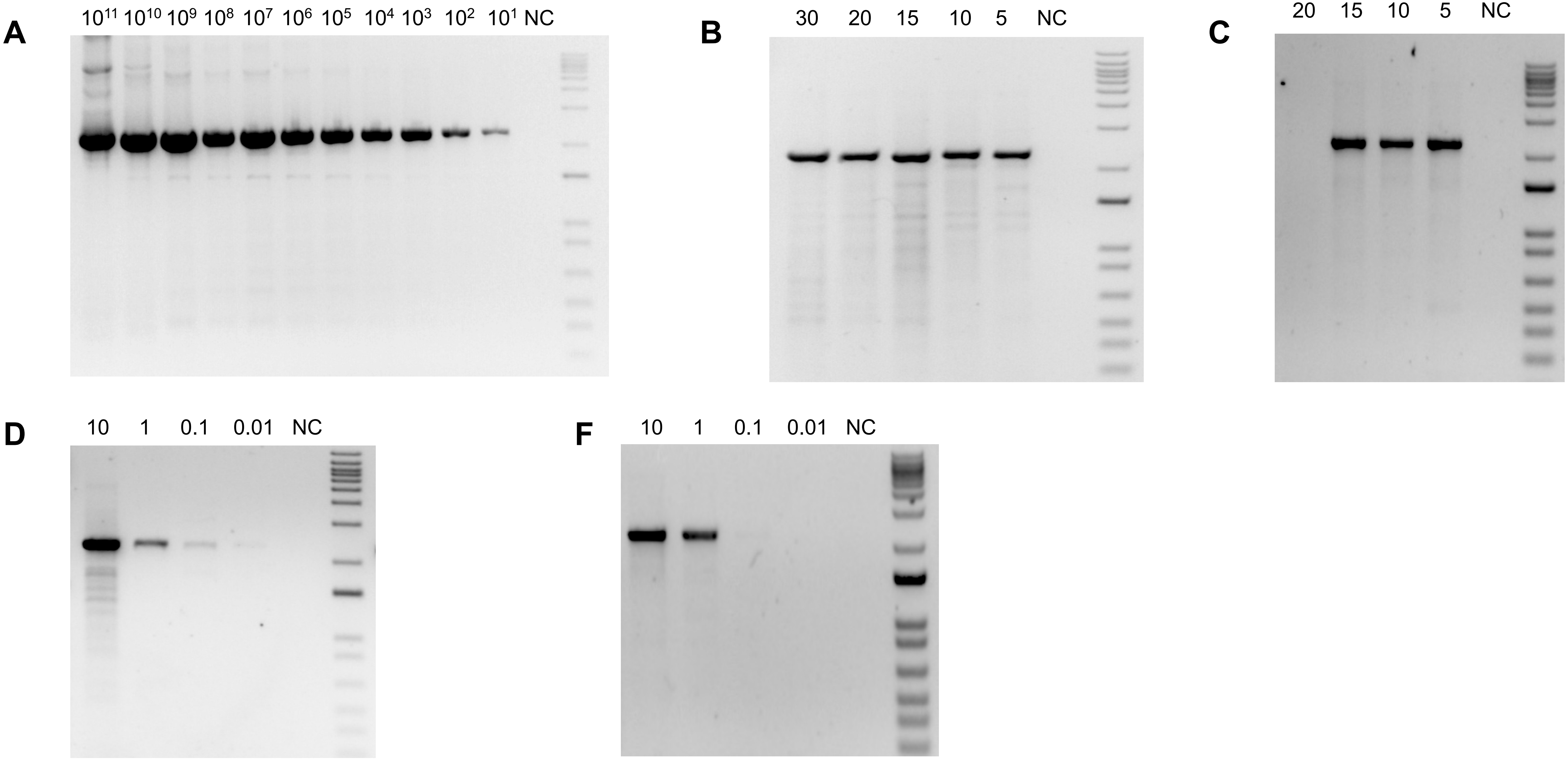

Supplementary Figure 3: A) Representative agarose gel image demonstrating minimum copy number required for PCR amplification using purified DNA (B) Representative agarose gel image demonstrating inhibitory concentration for PCR amplification using blood lysate as PCR template. C) Representative agarose gel image demonstrating inhibitory concentration for PCR amplification using buccal swab lysate as template D) Representative gel image demonstrating minimum concentration for PCR amplification using blood lysate as template. E) Representative gel image demonstrating minimum concentration for PCR amplification using buccal swab lysate

Supplementary Fig. 4

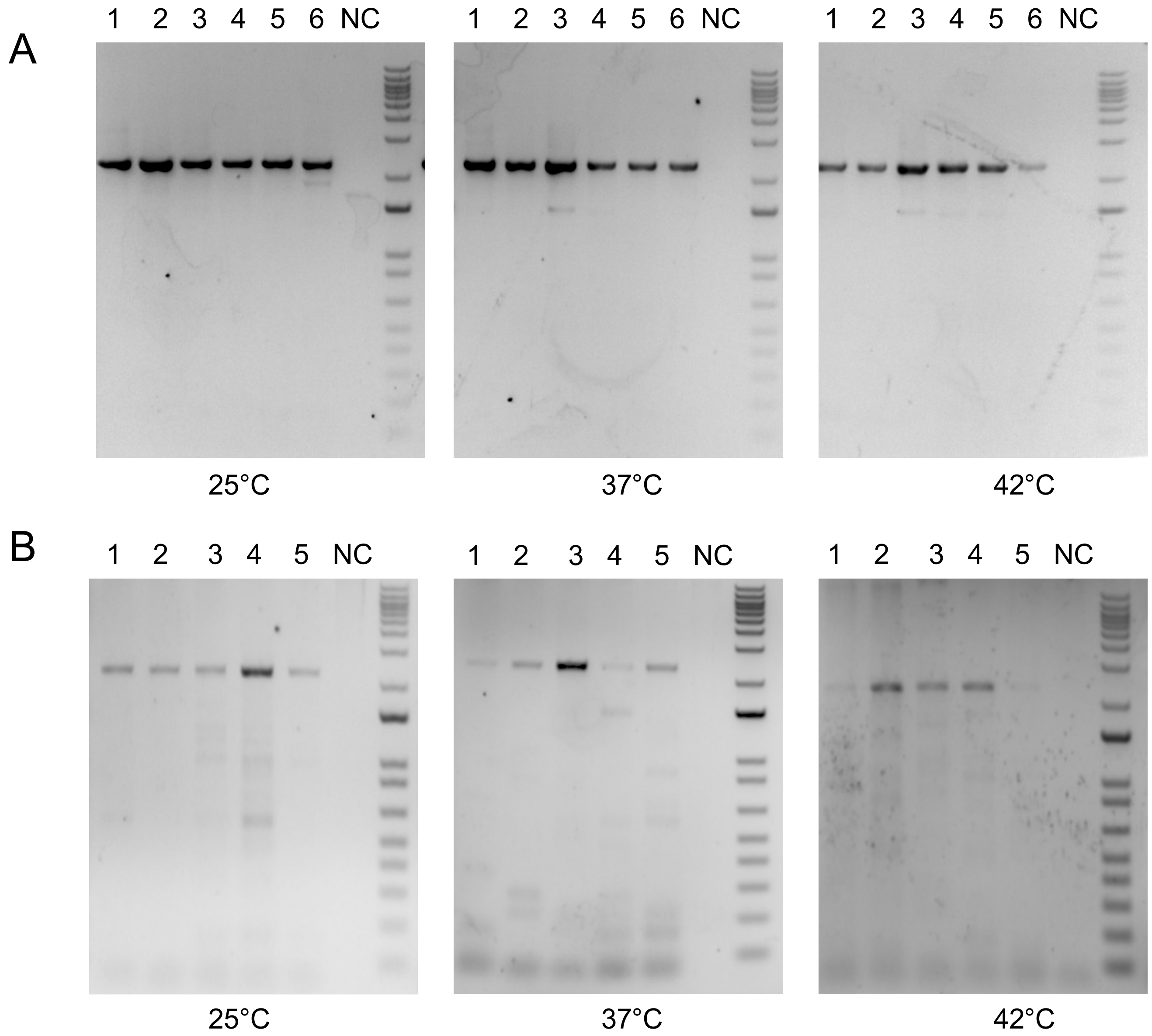

Supplementary Figure 4: A) Agarose gel images demonstrating the PCR amplification using buccal swab lysates stored at 25°C, 37°C and 42°C for 5 days before processing, n=6. **B)** Gel images demonstrating the PCR amplification using blood lysates stored at 25°C, 37°C and 42°C for 5 days as dry spots before processing, n=5.
